# Physiological alteration, quality of anesthesia and economy of isoflurane in domestic chickens (*Gallus domesticus)*

**DOI:** 10.14202/vetworld.2017.493-497

**Published:** 2017-05-08

**Authors:** Parag Deori, Kushal Konwar Sarma, Parsha Jyoti Nath, Chandan Kumar Singh, Rita Nath

**Affiliations:** 1 Department of Surgery and Radiology, College of Veterinary Science, Assam Agricultural University, Khanapara Campus, Guwahati, Assam, India; 2 Department of Veterinary Biochemistry, College of Veterinary Science, Assam Agricultural University, Khanapara Campus, Guwahati, Assam, India

**Keywords:** anesthetic quality, chicken (*Gallus domesticus*), economy, isoflurane, physiological parameters

## Abstract

**Aim:**

Aim of the study was to evaluate the effect of isoflurane anesthesia on physiological parameters, assessment of anesthetic qualities, and economy of use of isoflurane in domestic chickens (*Gallus domesticus*).

**Materials and Methods:**

In this study, 18 apparently healthy adult domestic chickens were selected randomly and divided into three groups. The birds were anesthetized by masked induction with isoflurane at a dose rate of 3.5%, 4%, and 5% and were maintained with 1.5%, 2%, and 2.5% isoflurane with oxygen by endotracheal intubation in Groups I, II, and III, respectively. Physiological parameters, viz., cloacal temperature, heart rate, and respiration rate were recorded at 0, 5, 10, 20, 30, 40, 50, and 60 min. The quality of anesthesia was assessed on the basis of induction time, induction behavior, quality of sedation, production of analgesia, degree of muscle relaxation, palpebral reflex, recovery time, and recovery behavior. The economy of anesthesia was calculated in terms of quantity of isoflurane utilized during 60 min of study. Statistical analysis was performed by analysis of variance, Duncan’s multiple range tests.

**Results:**

There was significant decrease (p<0.01) in physiological parameters such as in cloacal temperature, heart rate and respiration rate in the birds of all the groups from 0 to 60 min. The induction time was 5.83±0.33, 2.37±0.18, and 0.87±0.15 min, respectively, in Groups I, II, and III. Induction behavior was smooth in Group III, whereas mildly stormy in Group II and I. Quality of sedation was excellent in Group III, better in Group II as compared to Group I. Analgesia was moderate in Group III whereas poor in Group II and I. Degree of muscle relaxation was excellent in Group III, whereas good in Group I and II. Palpebral reflexes were absent in all the groups. Recovery time was 15.33±0.84, 18.83±0.94, and 26.50±0.85 in Groups I, II, and III respectively. Recovery behavior was smooth in birds of all the groups. The cost of the anesthesia was 158.22±1.04, 194.27±0.66, and 236.84±0.60 Indian National Rupee in Groups I, II, and III, respectively. Quantity of anesthesia utilized in each group was 7.62±0.05, 9.35±0.03, and 11.41±0.03 ml in Groups I, II, and III, respectively.

**Conclusion:**

The use of isoflurane at different concentration produces different level of physiological changes, quality of anesthesia and economy without causing any deleterious effect on the birds. The physiological parameters observed in this study can serve as reference values for the wild and endangered birds.

## Introduction

In avian, inhalational anesthetic is frequently used for physical examination, diagnostic procedures, surgical intervention, restrain, and research purposes [1]. The uses of anesthetics in birds have indicated that the tolerance of birds to most anesthetics between the surgical plane of anesthesia and death is relatively narrow [2]. Extreme care should be exercised when anesthetics are administered either by an inhalation or by an injectable route [2]. The objective of anesthesia is to maintain the possible lowest level of anesthetic to obtain restrain, to minimize stress, to mitigate pain nociception, and to induce muscle relaxation [3].

Isoflurane is the anesthetic of choice for birds [4,5] due to its relative safety and effectiveness [6], better control over the change of the anesthetic depth and recovery [7] and because of its rapid reversal resulting in a wide margin of safety and it is well tolerated by all the species [8]. Isoflurane does not produce any clinically deleterious effect on cardiorespiratory functions in pigeons [9]. Sevoflurane and desflurane [1] have some advantages over isoflurane but are comparatively costlier and thus their uses are limited in veterinary avian practices [4]. However, no anesthetic agent available till date is devoid of any biological impact.

This study was undertaken to evaluate physiological alteration, anesthetic quality and economy of use of isoflurane anesthesia on domestic chickens (*Gallus domesticus*) in the Indian condition, as it can serve as a reference for wild endangered species of birds.

## Materials and Methods

### Ethical approval

The study was approved by Institutional Animal Ethics Committee, Assam Agricultural University, College of Veterinary Science, Khanapara, Guwahati, 781022, Assam, India.

### Experimental program

The study was conducted in Department of Surgery and Radiology, College of Veterinary Science, Khanapara, Assam Agricultural University, on 18 healthy adult domestic chickens (*G. domesticus)* weighing 1.5-2 kg. The birds were obtained from Instructional Poultry Farm of the same institute. All the birds were acclimatized under the same managerial condition, feeding, watering, deworming for 2 weeks before study. The birds were prepared by withdrawing food for 4 h before anesthetic procedure; however, water was provided uninterruptedly. After the accomplishment of the procedure, the birds were released back to the instructional poultry farm after 48 h.

These birds were randomly divided into three groups comprising six birds in each group. Groups I, II, and III birds were induced with isoflurane at 3.5%, 4%, and 5% followed by endotracheal intubation after complete relaxation of beak and maintained with 1.5%, 2%, and 2.5%, respectively, with isoflurane in oxygen.

The physiological parameters recorded were cloacal temperature (°C), heart rate (beats/min), and respiration rate (breaths/min by inflation and deflation of re-breathing bag) at 0, 5, 10, 20, 30, 40, 50 and 60 min.

The quality of anesthesia was assessed on the basis of induction time from administration of isoflurane by masking till the bird became calm and quiet and showed no response to painful stimuli on the digits and central foot pad. Induction behavior was judged by grading as smooth/mildly stormy/stormy on the basis of loss of consciousness, abolition of reflex of beak, tongue, ease of endotracheal intubation, coughing and reaction if any by the bird.

Quality of sedation was graded as poor/good/excellent by signs of sedation such as ataxia, muscle relaxation, sternal or lateral recumbency, lowering of head and closing of eyelid. Production of analgesia was graded as poor/good/excellent by feather plucking and needle pricking at footpad. Degree of muscle relaxation was graded as poor/good/excellent on the basis of relaxation of beak, progressive decline in tonicity of muscles, relaxation of wings, and pedal reflex. Palpebral reflex was graded as present/sluggish/absent on the basis of touching the palpebra near medial canthus.

Recovery time was recorded as time from discontinuation of anesthesia to standing of the birds of its own and unaided without provocation. Recovery behavior was graded as smooth/moderate/stormy on the basis of shivering, shaking of head, blinking of eye, increased or decreased heart rate and respiration rate. The quality of recovery was scored as “excellent” with sternal position with little or no struggle; walked without assistance or struggle; once standing, did not fall to sternal recumbency; minimal ataxia when walking; as “satisfactory” with sternal position with little or no struggle; premature standing without weakness in hind limbs; once standing, fall to sternal recumbency unlikely; slight ataxia and finally assumed “poor” with some struggling; repeated attempts to move from lateral to sternal recumbency; premature standing with splayed and weak hind limbs; once standing, repeatedly falls to sternal recumbency; manual restraint required to avoid injury [10].

The economy of anesthesia was calculated in terms of quantity of isoflurane utilized during 60 min of study and calculated on Indian National Rupee (INR).

### Statistical analysis

The quantitative estimates of physiological and anesthetic parameters were analyzed as per SAS 9.3 package by performing Duncan multiple range test and analysis of covariance.

## Results and Discussion

In birds, anesthetic plane assessment on the basis of outward signs are difficult [11], however the depth of anesthesia can be evaluated by combining the information obtained from heart rate, respiration rate and reflexes [12-15]. Signs of anesthetic depth are dependent on the anesthetic used and vary among the different species [11]. Anesthetic depth signs vary from individuals to individuals, moments to moments during a single episode of anesthetics due to events like surgical stimulation and body temperature [11].

**Figure-1:**
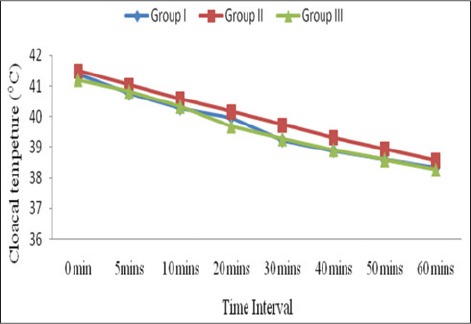
Mean cloacal temperature (°C) in birds of different groups.

In all the groups, there was a significant decrease (p<0.01) in cloacal temperature from 41.40±0.08°C to 38.35±0.19°C, 41.50±0.16°C to 38.55±0.19°C, and 41.20±0.08°C to 38.28±0.16°C from 0 to 60 min during anesthesia in Groups I, II, and III, respectively (Figure-1). Hypothermia associated bradyarrhythmia was not noted, the findings correlate [16]. The decrease in cloacal temperature during anesthesia may be due to disruption of thermoregulation and heat loss [17]. Decrease in cloacal temperature during isoflurane anesthesia have been was reported in crested serpent eagle (*Spilornis cheela hoya*) [16], red-tailed hawk (*Buteo jamaicensis*) [1], crested caracaras [5], chicken [18], and pigeon [19].

**Figure-2:**
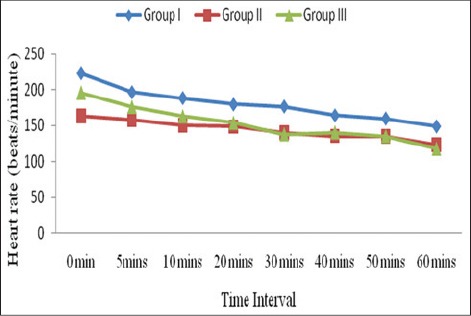
Mean heart rate (beats/min) in birds of different groups.

In the birds of Groups I, II and III, the heart rate was reduced significantly (p<0.01) from 223.33±15.02 to 149.00±16.06, 163.33±16.4 to 122.00±3.86, and 196.17±16.64 to 117.00±6.98 beats/min, respectively, from 0 to 60 min (Figure-2). Decrease in heart rate in all groups may be due to the depressant action of anesthetics on heart and myocardial function [8]. Although heart rate decreased significantly in all the groups, it was within the normal physiological limit and depicting minimum interference of isoflurane on cardiac functions. The decrease in heart rate during anesthesia were reported in bald eagle [20], chicken [21,22], hispaniolan amazon parrot *(Amazona ventralis)* [14], and ostriches [23]. Whereas, nonsignificant reduction in heart rate during anesthesia was reported in pigeon [19] and crested caracaras [5]. In wild red kite (*Milvus milvus*), heart rate was maintained at 240-260 beats/min during coelioscopy under isoflurane anesthesia [24]. No significant changes of heart rate were observed in cinereous vulture [25].

**Figure-3:**
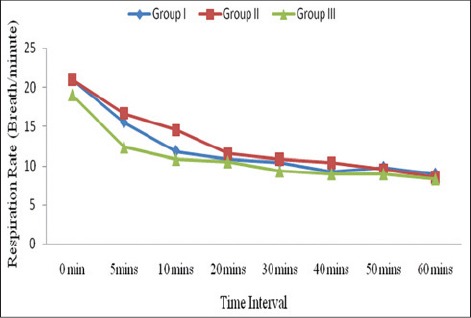
Mean respiratory rate (breaths/min) of birds indifferent groups.

Respiration rate was significantly decreased (p<0.01) from 21.00±15.20 to 9.00±1.12, 21.00±0.85 to 8.50±0.43, and 19.00±0.86 to 8.33±0.21 breaths/minute from 0 to 60 min during anesthesia (Figure-3). Decrease in respiratory rate might have resulted due to respiratory depressant activity of isoflurane further inducing hypercapnia [16]. These findings were concurrent to Hispaniolan amazon parrot (*A. ventralis*) [14], chicken [18], ostriches [23], crested caracaras [5]. However, respiration did not show a significant difference in pigeon during isoflurane anesthesia [19].

The mean±standard error (SE) of induction time (min) in Groups I, II, and III was 5.83±0.33, 2.37±0.18, and 0.87±0.15, respectively. The mean±SE of induction time is depicted in Table-1. Highly significant difference (p<0.01) in time of induction was observed between the Groups I, II and III. Faster induction was observed in Group III (0.87±0.15 min). The faster induction might be due to higher concentration of isoflurane delivered during induction.

**Table-1:** Mean±SE of induction time, recovery time, quantity of anesthesia, cost of anesthesia of individual birds.

Bird No.	Group I	Group II	Group III
Induction time (minutes)			
1	5.00	2.00	0.50
2	5.50	2.75	1.00
3	7.00	3.00	0.75
4	6.50	2.5	0.50
5	6.00	2.00	1.50
6	5.00	2.00	1.00
Mean±SE	5.83±0.33	2.37±0.18	0.87±0.15
Recovery time (minutes)			
1	15.00	20.00	30.00
2	17.00	19.00	25.00
3	12.00	22.00	28.00
4	18.00	15.00	25.00
5	15.00	18.00	26.00
6	15.00	19.00	25.00
Mean±SE	15.33±0.84	18.83±0.94	26.50±0.85
Quantity of anesthesia required (ml)			
1	7.50	9.30	11.34
2	7.56	9.41	11.43
3	7.80	9.45	11.39
4	7.72	9.37	11.34
5	7.65	9.30	11.53
6	7.50	9.30	11.44
Mean±SE	7.62±0.05	9.35±0.03	11.41±0.03
Cost of anesthetics (INR)			
1	155.62	192.97	235.38
2	157.18	195.31	237.33
3	161.85	196.9	236.35
4	160.29	194.53	235.38
5	158.74	192.97	239.27
6	155.62	192.97	237.33
Mean±SE	158.22±1.04	194.27±0.66	236.84±0.60

SE: Standard error

The quality of induction of anesthesia was smooth in all birds of Groups I, II and III. However, one bird of Group I showed stormy behavior during induction. Smooth and rapid induction in isoflurane anesthesia was reported in pigeon [19].

In Group I, 50% of birds had poor sedation and 50% had good sedation. All birds in Group II had good sedation while one of the birds had excellent sedation. In Group III, excellent sedation was observed in all the birds. The variation in quality of sedation in between the groups can be attributed to the difference in the delivery of concentration of the anesthetic. Good sedation quality of isoflurane anesthesia has been reported in bald eagles [20].

Analgesia was poor in Groups I and II whereas moderate in Group III. Moderate analgesia in Group III may be due to deep sedation. This might be because of the property of isoflurane that does not change the response threshold to noxious stimulation [26].

The degree of muscle relaxation in the birds of Group III was excellent while in Group II was good and Group I was poor. Excellent qualities of muscle relaxation in Group III might be due to the higher concentration of isoflurane anesthesia. A good muscle relaxation quality of isoflurane in ostriches has been reported [23]. Palpebral reflex was absent in all the birds of Groups I, II, and III.

The mean±SE of time of recovery from anesthesia was 15.33±0.84, 18.83±0.94, and 26.50±0.85 min in Groups I, II, and III, respectively. The mean±SE of recovery time is shown in Table-1. There was a highly significant difference (p<0.01) between the time of recovery in all the groups. The prolonged recovery from anesthesia in Group III might be due to the higher concentration of isoflurane delivered in the birds of this group and higher degree of central nervous system depression.

The birds in Group I exhibited short duration of sedation with light analgesia and muscle relaxation. The birds showed struggling, repeated attempts to move from lateral to sternal recumbency, stumbling and required manual assistance during recovery. On the other hand, the birds in Group II, recovery was found to be without struggle, premature standing without weakness in the hind limbs and occasional slight ataxia; while in Group III, the bird showed sternal position with no struggle, walk without assistance, minimal ataxia when walking and did not fall to sternal recumbency during recovery period. Similar findings were reported in ostriches with isoflurane anesthesia during recovery [23].

The mean±SE of quantity of anesthesia (ml) required was 7.62±0.05, 9.35±0.03, and 11.41±0.03 in Groups I, II III, respectively. The mean±SE of quantity of anesthesia (ml) is depicted in Table-1. There was highly significant difference (p<0.01) between the quantities of anesthesia required in birds of all groups. The quantity of anesthesia required was highest in Group III in comparison to other groups. This was due to highest concentration of isoflurane used in Group III.

The average calculated cost of anesthesia (INR) was 158.22±1.04, 194.27±0.66, and 236.84±0.60 in Groups I, II and III, respectively. The mean±SE of cost of anesthesia (INR) is shown in Table-1. There was a highly significant difference (p<0.01) between the costs of anesthesia in all groups. The cost of anesthesia was highest in Group III in comparison to other groups. This was due to the highest quantity of isoflurane used in Group III.

No complications were observed in birds of all three groups at different concentration of isoflurane used in this study.

## Conclusion

Isoflurane produced anesthesia of varying degree in the domestic chicken (*G. domesticus*) on the basis of their concentration used. These concentration ranges of the anesthetic used showed different level of effect on the physiological parameters, anesthetic qualities without any adverse effect during recovery. The different levels of anesthetic concentration used have varied impact on the economy. It can be concluded that 5% induction and 2.5% maintenance of isoflurane in oxygen was found to produce ideal level of surgical anesthesia in *G. domesticus*.

## Authors’ Contributions

This study was a part of PD’s research work during his M.V.Sc. program. PD, KKS, PJN and RN have conceived, planned and designed the study. PD, PJN, CKS and RN have conducted the research, analyzed and kept a due record of the data. Manuscript was framed and drafted by PD and CKS under the aegis of KKS. All authors read and approved the final manuscript.
